# Supplementary material for: Updated Guidance for Communicating PFAS Identification Confidence with Ion Mobility Spectrometry
Source: bioRxiv. 2025 May 17:2025.01.27.634925. Originally published 2025 Jan 28. Preprint. [Version 2] doi: 10.1101/2025.01.27.634925 (PMC11838322; doi:10.1101/2025.01.27.634925)
Supplement: Supplement 1 [file media-1.pdf]

LC- or GC-IMS-HRMS Confidence Level Requirement Checklist for PFAS

All boxes must be checked for PFAS identifications at each level. Explicit match criteria, tolerances, experimental values and spectra, and specific diagnostic evidence should be reported for each identification.

Confirmed Structure (Level 1)

Exactly one structure confirmed by reference standard analyzed under identical conditions

- ☐ Exactly one structure
- ☐ Reference standard analyzed in house using same analytical methods
- ☐ Exact *m/z* matches reference standard
- ☐ Isotopic pattern matches reference standard
- ☐ CCS matches reference value
- ☐ RT or RI matches reference value
- ☐ One or both of:
  - Mobility-aligned fragments match reference spectrum
  - Diagnostic evidence

Probable Structure (Level 2)

Exactly one structure with sufficient evidence to rule out all other structural possibilities

- ☐ Exactly one structure
- ☐ Exact *m/z* matches structure
- ☐ Isotopic pattern matches structure
- ☐ CCS matches library or predicted value
- ☐ RT or RI matches library or predicted value
- ☐ One or both of:
  - Mobility-aligned fragments match library or predicted spectrum
  - Diagnostic evidence

Tentative Candidate Structure(s) (Level 3)

One or more candidate structures satisfy all experimental evidence

- ☐ One or more structures
- ☐ Exact *m/z* matches all candidates
- ☐ Isotopic pattern supports all candidates
- ☐ CCS library or predicted value supports all candidates
- ☐ RT or RI library or predicted value supports all candidates
- ☐ One or both support all candidates:
  - Mobility-aligned fragments
  - Diagnostic evidence

Unequivocal Formula (Level 4)

Exactly one molecular formula is possible based on all available evidence

- ☐ Exactly one possible formula
- ☐ Exact *m/z* matches formula
- ☐ Isotopic pattern supports formula
- ☐ One or both support formula:
  - Mobility-aligned fragments
  - Diagnostic evidence

Feature of Interest (Level 5)

At least one supporting dimension of data indicating the feature is of interest

- ☐ One or more of:
  - Exact *m/z* matches a suspect list PFAS
  - Mass defect is indicative of fluorine atoms
  - CCS vs *m/z* falls in fluorinated space
  - Other justifiable attributes not listed here

Definitions

- Match: the measured value is within user-defined acceptable error tolerance of a comparison value
- Support: the measured attribute provides evidence that aligns with the proposed structures or formula
- LC: liquid chromatography
- GC: gas chromatography
- IMS: ion mobility spectrometry
- HRMS: high resolution mass spectrometry
- PFAS: per- and polyfluoroalkyl substances
- CCS: collision cross section (obtained from ion mobility separation)
- RT: chromatographic retention time
- RI: chromatographic retention index (typically applicable to GC only)
- Diagnostic evidence: other experimental data supporting the PFAS identification, including but not limited to relevant synthesis information, geographic knowledge, presence of homologues, and parent-transformation product relationships
- Homologue: CF_2 or other molecular repeating unit found in known PFAS structures
