# Supplementary material for: Updated Guidance for Communicating PFAS Identification Confidence with Ion Mobility Spectrometry
Source: bioRxiv. 2025 May 17:2025.01.27.634925. Originally published 2025 Jan 28. Preprint. [Version 2] doi: 10.1101/2025.01.27.634925 (PMC11838322; doi:10.1101/2025.01.27.634925)
Supplement: Supplement 2 [file media-2.pdf]

Supplemental information for

Updated Guidance for Communicating PFAS Identification Confidence with Ion Mobility Spectrometry

Anna K. Boatman[1], Jessie R. Chappel[2-4], Kaylie I. Kirkwood-Donelson [5], Jonathon F. Fleming[6], David M. Reif[6], Emma L. Schymanski[6*], Julia E. Rager[2-4*], and Erin S. Baker[1*]

1. Department of Chemistry, University of North Carolina at Chapel Hill, Chapel Hill, North Carolina 27514, USA
2. Department of Environmental Sciences and Engineering, Gillings School of Global Public Health, The University of North Carolina at Chapel Hill, Chapel Hill, North Carolina 27514, USA
3. The Institute for Environmental Health Solutions, Gillings School of Global Public Health, The University of North Carolina at Chapel Hill, Chapel Hill, North Carolina 27514, USA
4. Curriculum in Toxicology and Environmental Medicine, School of Medicine, University of North Carolina, Chapel Hill, North Carolina 27514, USA
5. Immunity, Inflammation, and Disease Laboratory, National Institute of Environmental Health Sciences, Durham, North Carolina 27709, USA
6. Predictive Toxicology Branch, Division of Translational Toxicology, National Institute of Environmental Health Sciences, Durham, North Carolina 27713, USA
7. Luxembourg Centre for Systems Biomedicine (LCSB), University of Luxembourg, 6 Avenue du Swing, L-4367, Belvaux, Luxembourg
   * Correspondence: Emma L. Schymanski: emma.schymanski@uni.lu, Julia E. Rager: jrager@unc.edu, Erin S. Baker: erinmsb@unc.edu

Summary: 10 pages, 3 figures, 4 tables

Contents

Table S1 .... S3
Table S2 .... S5
Figure S1 .... S6
Table S3 .... S8
Table S4 .... S9
Figure S3 .... S10

Table S1. The 38 papers on PFAS NTA published in 2023 and included in the in-depth analysis.

Study ID	PMID	Chromato-graphy used	MS platform used in NTA	IMS used	Matrix category	Experiment category	Confidence levels reported?	Count of PFAS detected with HRMS	1	2	2a	2bc	3	4	5
3	36622151	LC	Orbitrap	No	Water	PFAS-environmental	Yes-Schymanski	18	0	18	0	18	0	0	0
28	36459821	GC	TOF	Yes-cIMS	Multiple	PFAS-environmental	No	25	na	na	na	na	na	na	na
29	36577148	LC	Orbitrap	No	Standards	PFAS-remediation	No	52	na	na	na	na	na	na	na
7	36827766	LC	Orbitrap	No	Water	PFAS-environmental	Yes-Schymanski	54	33	16	na	na	5	0	0
8	37058300	LC	TOF	No	Soil	PFAS-environmental	Yes-Charbonnet	87	0	87	0	87	0	0	0
9	37100129	LC	TOF	No	Blood	PFAS-environmental	Yes-Schymanski	93	0	28	0	0	0	0	0
27	36787488	Multiple	Multiple	No	Other industrial	Pollutants-industrial	Yes-Schymanski	0	na	na	na	na	na	na	na
6	36801580	LC	Orbitrap	No	Water	PFAS-environmental	Yes-Charbonnet	30	17	7	1	6	6	0	0
17	37648245	LC	TOF	No	Blood	PFAS-environmental	Yes-Charbonnet	30	13	2	2	0	14	0	1
30	36863195	GC	Orbitrap	No	Standards	PFAS-other	Yes-other	na	na	na	na	na	na	na	na
31	36947591	GC	Orbitrap	No	Air	PFAS-industrial	No	5	na	na	na	na	na	na	na
19	37800548	Multiple	Multiple	No	Multiple	PFAS-environmental	Yes-Schymanski	82	32	13	na	na	12	11	0
20	37816443	LC	Orbitrap	No	Water	PFAS-environmental	Yes-Schymanski	50	0	13	na	na	27	10	0
32	37196788	LC	TOF	No	Water	PFAS-remediation	No	15	na	na	na	na	na	na	na
23	37923386	LC	Orbitrap	No	Water	PFAS-environmental	Yes-Schymanski	44	0	0	0	0	1	0	43
33	37207413	LC	TOF	No	Multiple	PFAS-environmental	No	2	na	na	na	na	na	na	na
25	37991390	LC	Orbitrap	No	Soil	PFAS-environmental	Yes-Schymanski	67	47	3	na	na	15	2	0
12	37307429	SFC	TOF	No	Water	Pollutants-environmental	Yes-Schymanski	3	3	0	0	0	0	0	0
13	37394749	LC	Orbitrap	No	Blood	Pollutants-environmental	Yes-Schymanski	21	4	0	na	na	na	na	na

14	37414869	LC	Orbitrap	No	Air	Pollutants-environmental	Yes-Schymanski	2	1	0	na	na	1	0	0
1	36341487	LC	TOF	No	Water	PFAS-environmental	Yes-Charbonnet	47	4	18	12	6	12	4	9
16	37590049	LC	Orbitrap	No	Other industrial	PFAS-industrial	Yes-Charbonnet	24	0	0	0	0	6	4	3
15	37419156	LC	TOF	No	Other industrial	PFAS-industrial	Yes-Schymanski	3	0	3	3	0	0	0	0
18	37723602	LC	TOF	No	Blood	PFAS-environmental	Yes-Schymanski	64	16	12	na	na	3	33	0
2	36374630	None	FTICR	No	Water	PFAS-remediation	Yes-Schymanski	68	0	5	na	na	0	63	0
4	36722905	LC	Orbitrap	No	Standards	PFAS-remediation	Yes-Charbonnet	28	0	13	0	13	12	0	1
34	37856848	LC	Orbitrap	No	Other industrial	PFAS-remediation	No	37	na	na	na	na	na	na	na
21	37878714	LC	TOF	Yes-DTIMS	Water	PFAS-environmental	Yes-Charbonnet	57	36	8	3	5	3	4	6
22	37878998	LC	Orbitrap	No	Water	PFAS-environmental	Yes-Schymanski	26	0	26	0	26	0	0	0
35	37884429	LC	Not specified	No	Blood	PFAS-environmental	No	18	na	na	na	na	na	na	na
10	37200532	LC	TOF	No	Standards	PFAS-remediation	Yes-Charbonnet	102	0	33	8	25	11	0	47
36	37934628	LC	TOF	No	Other biological	Pollutants-environmental	Yes-other	3	na	na	na	na	na	na	na
24	37970096	LC	Orbitrap	No	Multiple	PFAS-industrial	Yes-Schymanski	22	1	9	na	na	10	2	0
37	37988936	GC	TOF	No	Multiple	PFAS-environmental	No	10	na	na	na	na	na	na	na
5	36736215	LC	Orbitrap	No	Water	Pollutants-environmental	Yes-Schymanski	15	0	0	0	0	1	0	0
26	38133352	LC	TOF	No	Multiple	PFAS-environmental	Yes-Schymanski	3	1	0	0	0	0	0	1
11	37222023	LC	Orbitrap	No	Blood	Pollutants-environmental	Yes-Schymanski	11	0	3	0	0	0	0	0
38	37503704	Multiple	TOF	No	Other industrial	PFAS-industrial	No	0	na	na	na	na	na	na	na

Table S2. Breakdown of types of experiments from 38 papers on PFAS NTA published in 2023.

**Analytical platform**					
**HRMS instrument**	**Count**	**Chromatography**	**Count**	**IMS**	**Count**
Orbitrap	18	LC	29	None	36
TOF	16	GC	4	cIMS	1
Multiple	2	Multiple	3	DTIMS	1
FTICR	1	SFC	1		
Not specified	1	None	1		
**Experiment type**					
**Molecules analyzed**		**Matrix analyzed**			
**PFAS specific**	**Count**	**Matrix category**	**Count**	**Water type**	**Count**
Environmental	19	Water	12	Wastewater	5
Remediation	6	Blood	6	Ground water	4
Industrial	5	Multiple	6	Surface water	3
Other	1	Other	6	Drinking water	1
**Pollutants (including PFAS)**	**Count**	Standards	4	Sea water	1
Environmental	6	Soil	2	Passive samplers	1
Industrial	1	Air	2		

Definitions
HRMS: high resolution mass spectrometry
TOF: time of flight
FTICR: Fourier transform ion cyclotron resonance
LC: liquid chromatography
GC: gas chromatography
SFC: supercritical fluid chromatography
IMS: ion mobility spectrometry
cIMS: cyclic IMS
DTIMS: drift tube IMS

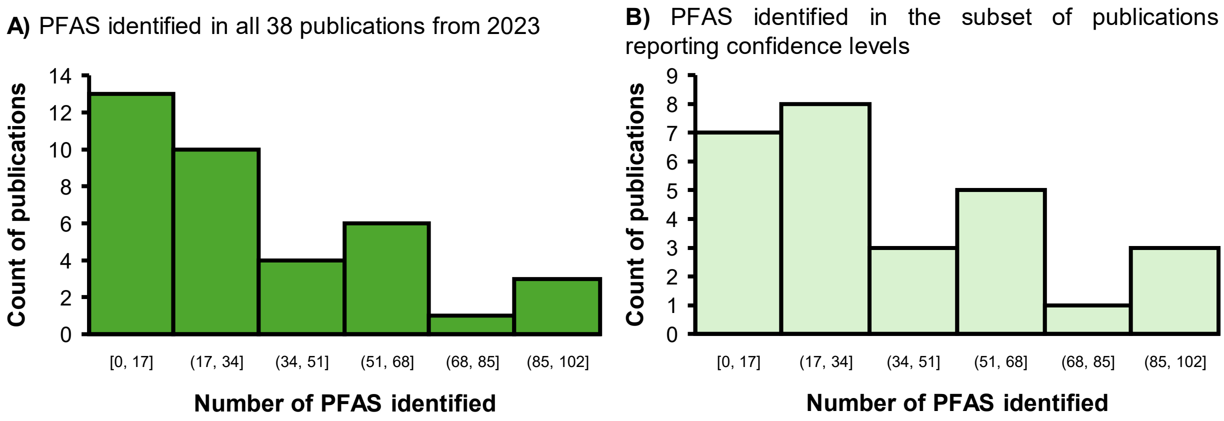

Figure S1. **A)** Number of PFAS identified in papers on PFAS NTA published in 2023 in 37 of the 38 papers evaluated (one of the 38 papers evaluated did not report a number of PFAS identified and is not included here). Min = 0, max = 102, median = 25, average = 32.3 PFAS identified per study. **B)** The subset of 27 papers that also reported a confidence level on a 1-5 scale. Min = 0, max = 102, median = 30, average = 38.1 PFAS identified per study.

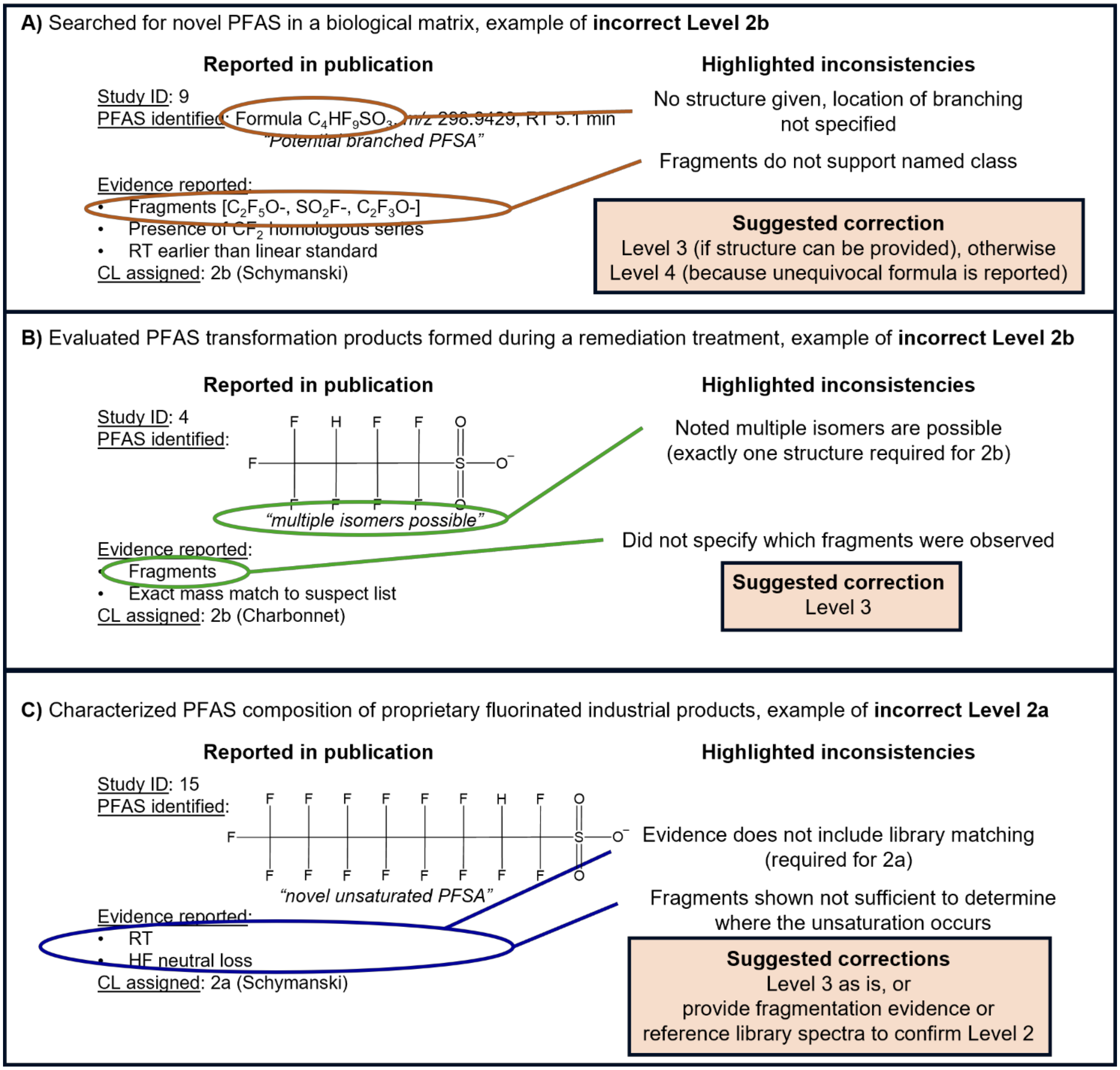

Figure S2. Examples of confidence levels assigned in a manner inconsistent with the scale cited by each study. **A)** This study cited the Schymanski scale and assigned a Level 2b to a molecule reported as a "potential branched PFSA"; however, no structure was provided and the reported fragments do not match with the implied structure for a branched PFSA (a fully fluorinated carbon chain with a sulfonic acid head group). **B)** This study cited the Charbonnet confidence scale, assigned a Level 2b to a structure for an unsaturated PFSA, and noted "multiple isomers possible". Fragmentation evidence could potentially distinguish between different isomers; however, if multiple isomers were observed it would be better to report all of them individually. **C)** This study cited the Schymanski scale and assigned a Level 2a to a structure referred to in the text as a "novel unsaturated PFSA" (author note: this could also be termed hydrogen substituted PFSA, but the name used in the original study has been retained for consistency). Level 2a requires spectral matching to a library and therefore it is highly unlikely that truly novel (i.e. previously undiscovered) structures could be elucidated in this manner. Insufficient evidence is provided to support a Level 2 assignment.

Table S3. Summary of proposed changes to confidence level guidance for PFAS identifications made using LC-IMS-HRMS

**Attribute**	**Current guidance**	**Proposed change**
Mobility-aligned fragments	MS^n "matching" library or reference spectra,[1,2] minimum 1, 2, or 3 diagnostic or subclass-aligned fragments[3]	Not required when diagnostic evidence is available
CCS value	Must match library CCS for Level 2a or predicted CCS for Level 2b or 3[2]	Library or predicted value is acceptable if within user-defined tolerance
RT value	Must match reference standard ≤ 0.1 min for Level 1, must match library for Level 2a[2]<br>Not required for homologous series at Level 2b or 3[3]	Library or predicted RT or RI value is acceptable if within user-defined tolerance
PFAS-specific sublevels	Confidence decreases in order: 1a > 1b > 2a > 2b and 2c > 3a > 3b > 3c and 3d > 4 > 5a and 5b[3]	Levels 1-5 only, no sublevels

Table References

(1) Schymanski, E. L.; Jeon, J.; Gulde, R.; Fenner, K.; Ruff, M.; Singer, H. P.; Hollender, J. Identifying Small Molecules via High Resolution Mass Spectrometry: Communicating Confidence. Environmental Science & Technology 2014, 48 (4), 2097-2098. DOI: 10.1021/es5002105.

(2) Celma, A.; Sancho, J. V.; Schymanski, E. L.; Fabregat-Safont, D.; Ibáñez, M.; Goshawk, J.; Barknowitz, G.; Hernández, F.; Bijlsma, L. Improving Target and Suspect Screening High-Resolution Mass Spectrometry Workflows in Environmental Analysis by Ion Mobility Separation. Environmental Science & Technology 2020, 54 (23), 15120-15131. DOI: 10.1021/acs.est.0c05713.

(3) Charbonnet, J. A.; McDonough, C. A.; Xiao, F.; Schwichtenberg, T.; Cao, D.; Kaserzon, S.; Thomas, K. V.; Dewapriya, P.; Place, B. J.; Schymanski, E. L.; et al. Communicating Confidence of Per- and Polyfluoroalkyl Substance Identification via High-Resolution Mass Spectrometry. Environmental Science & Technology Letters 2022, 9 (6), 473-481. DOI: 10.1021/acs.estlett.2c00206.

Table S4. Worksheet for defining platform-specific tolerances of each measured dimension. Suggested definitions are provided and users should modify the suggestions as appropriate.

Parameter	Match type	Tolerance definition
Exact *m/z*	All	Measured *m/z* is ± _________ of the exact *m/z*; same adducts observed as with the reference standard
Isotopic pattern	All	
CCS	Reference standard	Measured CCS is ± _____ of the reference standard value / in-house library value
	Library value	Measured CCS is ± _____ of external library value
	Predicted value	
RT or RI	Reference standard	Measured RT / RI is ± ______ of internal / external standards<br>Measured RT / RI is ± ______ of in-house library value from identical chromatographic method
	Library value	Measured RT is ± ______ of library value and must elute in the same order as other detected library matches
	Predicted value	
Mobility-aligned fragments	Reference standard	MS/MS match cosine similarity is > ________
	Library value	Same instrument: MS/MS match cosine similarity is > ________<br>Different instrument: MS/MS match cosine similarity is > ________

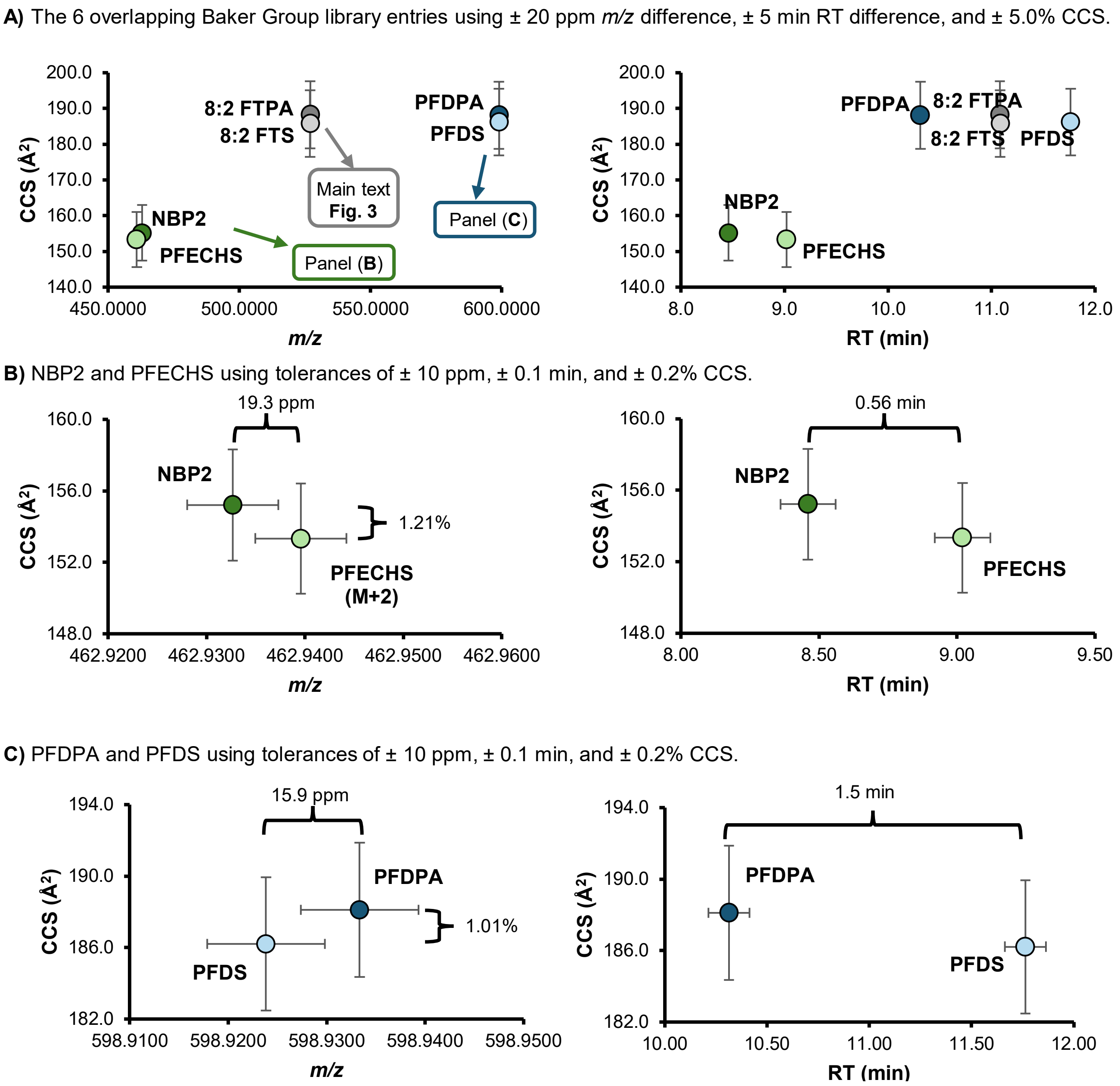

Figure S3. Evaluation of our LC-IMS-HRMS library revealed only 6 PFAS with the potential to overlap with another PFAS when considering *m/z* and CCS. CCS and RT values are from the Baker Group PFAS LC-IMS-MS library, v3, available on Zenodo at DOI: https://doi.org/10.5281/zenodo.13387492. **A)** All 6 PFAS in the library found to overlap (excluding branched isomers of PFOA and PFOS, which have been described elsewhere) using wide tolerances of ± 20 ppm *m/z* difference and +1 or +2 Da to account for isotopic envelopes, ± 5 min RT difference, and ± 5.0% CCS. **B)** Nafion Byproduct 2 (NBP2) and PFECHS are 19.3 ppm different and should typically resolve with HRMS alone. However, care should be taken especially if either peak is particularly abundant, because these molecules elute closely and their CCS values are also quite close (1.21%). MS2 may be advised for confirming these structures in some cases, depending on the tolerance of the instrument. **C)** PFDS and PFDPA are 15.9 ppm different in *m/z*, 1.01% different in CCS, and elute 1.5 min apart on our typical LC gradient. If using a different gradient or if matrix effects are observed, MS2 is recommended to confirm which structures are present in the sample.
