# Supplementary material for: Updated Guidance for Communicating PFAS Identification Confidence with Ion Mobility Spectrometry
Source: bioRxiv. 2025 Jan 28:2025.01.27.634925. Preprint. [Version 1] doi: 10.1101/2025.01.27.634925 (PMC11838322; doi:10.1101/2025.01.27.634925)
Supplement: Supplementary file 1 [file media-1.pdf]

LC- or GC-IMS-HRMS Confidence Level Requirement Checklist for PFAS
All boxes must be checked for PFAS identifications at each level

Confirmed Structure (Level 1)
Exactly one structure confirmed by reference standard analyzed under identical conditions

- ☐ Exactly one structure
- ☐ Reference standard analyzed in house using same analytical methods
- ☐ Exact *m/z* matches reference standard
- ☐ Isotopic pattern matches reference standard
- ☐ CCS matches reference value
- ☐ RT or RI matches reference value
- ☐ One or both of:
  - Mobility-aligned fragments match reference spectrum
  - Diagnostic evidence *e.g*. PFAS synthesis, homologues, parent-TPs

Probable Structure (Level 2)
Exactly one structure with sufficient evidence to rule out all other structural possibilities

- ☐ Exactly one structure
- ☐ Exact *m/z* matches structure
- ☐ Isotopic pattern matches structure
- ☐ CCS matches library or predicted value
- ☐ RT or RI matches library or predicted value
- ☐ One or both of:
  - Mobility-aligned fragments match library or predicted spectrum
  - Diagnostic evidence *e.g*. PFAS synthesis, homologues, parent-TPs

Tentative Candidate Structure(s) (Level 3)
One or more candidate structures satisfy all experimental evidence

- ☐ One or more structures
- ☐ Exact *m/z* matches all candidates
- ☐ Isotopic pattern supports all candidates
- ☐ CCS library or predicted value supports all candidates
- ☐ RT or RI library or predicted value supports all candidates
- ☐ One or both support all candidates:
  - Mobility-aligned fragments
  - Diagnostic evidence *e.g*. PFAS synthesis, homologues, parent-TPs

Unequivocal Formula (Level 4)
Exactly one molecular formula is possible based on all available evidence

- ☐ Exactly one possible formula
- ☐ Exact *m/z* matches formula
- ☐ Isotopic pattern supports formula
- ☐ One or both support formula:
  - Mobility-aligned fragments
  - Diagnostic evidence *e.g*. PFAS synthesis, homologues, parent-TPs

Feature of Interest (Level 5)
At least one supporting dimension of data indicating the feature is of interest

- ☐ One or more of:
  - Exact *m/z* matches a suspect list PFAS
  - Mass defect is indicative of fluorine atoms
  - CCS vs *m/z* falls in fluorinated space
  - Other justifiable attributes not listed here

Definitions

- Match: the measured value is within user-defined acceptable error tolerance of a comparison value
- Support: the measured attribute provides evidence that aligns with the proposed structures or formula
- LC: liquid chromatography
- GC: gas chromatography
- IMS: ion mobility spectrometry
- HRMS: high resolution mass spectrometry
- PFAS: per- and polyfluoroalkyl substances
- CCS: collision cross section obtained from ion mobility separation
- RT: chromatographic retention time
- RI: chromatographic retention index (typically applicable to GC only)
- Homologue: CF_2 or other molecular repeating unit found in known PFAS structures
- TP: transformation product
